# Highly Sensitive Immunochromatographic Detection of Porcine Myoglobin as Biomarker for Meat Authentication Using Prussian Blue Nanozyme

**DOI:** 10.3390/foods12234252

**Published:** 2023-11-24

**Authors:** Olga D. Hendrickson, Elena A. Zvereva, Boris B. Dzantiev, Anatoly V. Zherdev

**Affiliations:** A.N. Bach Institute of Biochemistry, Research Center of Biotechnology of the Russian Academy of Sciences, Leninsky Prospect 33, 119071 Moscow, Russia; odhendrick@gmail.com (O.D.H.); zverevaea@yandex.ru (E.A.Z.); dzantiev@inbi.ras.ru (B.B.D.)

**Keywords:** myoglobin, lateral flow immunoassay, nanozyme, Prussian blue, signal enhancement, meat authentication

## Abstract

This study was aimed at the sensitive immunodetection of porcine myoglobin (MG) as a species-specific biomarker in meat products. The enhanced lateral flow immunoassay (LFIA) was created in the sandwich format using monoclonal antibodies (Mab) with specificity to porcine MG and labeled by Prussian blue nanoparticles (PBNPs) as peroxidase-mimicking nanozymes. Signal amplification was provided by the colored product of oxidation catalyzed by the PBNPs. Several Mab–PBNP conjugates with different antibody loads were synthesized; the one that provided the best analytical characteristics of the LFIA was selected. Advanced optimization of the test system was carried out. As a result, the visual limit of detection (LOD) of MG was 1.5 ng/mL. Involvement of the catalytic nanozyme properties allowed the LOD to be decreased by ~9 times in comparison to the LFIA based on gold nanomarkers, and by ~27 times compared to the LFIA based on PBNP coloration. The assay time was 30 min, including catalytic enhancement. A simple technique of meat sample pre-treatment aimed at effective MG extraction and matrix disposal was proposed. The specificity of the LFIA towards the pork meat was demonstrated. The applicability of the created test system was shown by testing extracts obtained from finished meat products.

## 1. Introduction

The safety of food products is one of the fundamental factors in improving the quality of human life [1]. Providing the population with healthy nutrition is an integral part of the policy of caring for health and increasing life expectancy. Meat and meat-containing products are essential components of the diet of most people [2]. The quality of meat-based products is an important factor in determining their nutritional value and safety [3]. Despite the obvious need to comply with safety and quality standards for meat foodstuffs, the problem of counterfeiting is currently acute [4]. To increase profits and reduce production costs, manufacturers can use undeclared additives or plant analogs as the main raw materials and replace costly types of meat with cheaper substitutes [5]. The inadmissibility of certain meat types in some religious denominations (Islam, Judaism) and ethical issues associated with the ban on the consumption of some of them (mainly pork-based) are other concerns [6,7,8]. All this necessitates rigorous control of meat-product composition at all stages—from the processing of raw materials and production to the sale of finished products [9,10].

The tools to control the authenticity of meat products include the techniques of proteomics and peptidomics [11,12], molecular genetic methods [13,14,15], and different chromatographic approaches [16,17]. They are specific and sensitive but are performed by qualified operators using expensive equipment. The testing productivity for the majority of these methods is limited by the small number of samples analyzed simultaneously and cannot be increased without purchasing additional equipment, or in some cases, moving to very expensive automated high-throughput analyzers. In this context, methods providing not only sensitive and accurate but also rapid and simple on-site detection of target analytes are in great demand. Immunochromatographic test systems based on the principles of lateral flow separation by porous membranes and highly specific antigen–antibody interactions fully meet these requirements [18,19]. The advantages of the lateral flow immunoassay (LFIA) are rapidity (15–30 min), simplicity of the analytical procedure, low cost of tests, sensitivity, and specificity. In the simplest LFIA format, all reactants are anticipatorily applied to the test strip, and the analysis consists of incubating the strip with the tested sample. For more complex LFIA protocols, simultaneous testing of many samples is ensured by synchronization of multiple test strip immersion into prepared reagents or by the involvement of additional operators without the necessity of special training. The indicated features enable the use of LFIA for rapid screening of food samples in out-of-laboratory conditions.

To verify the composition of meat foodstuffs (a particular type of meat) by LFIA, it is important to choose a proper molecular identifier and an efficient marker. The identifier should be species-specific and contained in the target tissue (e.g., in muscles) in adequate concentrations. Furthermore, it should be extractable from the tissue in a non-destructive manner. Literature sources describe approaches to discriminate between meat sources using species-specific identifiers, such as immunoglobulins [20,21,22], troponin and myosin [23], porcine gelatin [24], hemoglobin [25], species-characteristic peptides [17,26], and some others. As an effective molecular biomarker for meat authentication, myoglobin (MG) can be considered, being a muscle tissue protein characterized by different amino acid sequences for different animal species [27,28]. LFIAs of MG are described as molecular markers in some studies [29,30,31], where the authors successfully implemented species recognition of the meat sources.

The sensitivity of the assay developed in model conditions is often insufficient to detect analytes in real samples. Therefore, there is great demand for approaches aimed at increasing the LFIA sensitivity to ensure accurate detection and eliminate false negative results [32]. An efficient approach is the amplification of the analytical zone coloration on the test strip. Traditionally, gold nanoparticles (AuNPs) are used as colorimetric markers to produce bright red bands [33,34,35,36]. However, recently, nanozymes, which are nanoparticles containing metal(s) and possessing enzyme-like catalytic activities [37,38,39] have become promising as markers for the LFIA. Compared to their natural analogs, nanozymes are characterized by high stability and easy and low-cost preparation, and may demonstrate higher activity due to multiple active sites. The use of nanozymes in immunosensors and immunoassays is summarized in some reviews [40,41,42], as is their use specifically for food control [43,44]. Several studies have shown that in nanozyme-based LFIAs with catalytic enhancement, the analyte’s limit of detection (LOD) can be reduced due to increased coloration in the bands of test strips [30,45,46,47].

In the current study, a highly sensitive immunochromatographic analysis for the enhanced determination of MG as a molecular biomarker of pork when monitoring the composition of meat products has for the first time been developed using Prussian blue nanoparticles (PBNPs) as a nanozyme label. Prussian blue is a mixture of hexacyanoferrates, which is obtained by the reaction of ferric salts with potassium hexacyanoferrate (II). Before the discovery of catalytic properties, blue-colored Prussian blue was used as a pigment. Later, Prussian blue found application as a peroxidase-mimic catalytic material for the selective reduction of hydrogen peroxide [47,48]. Due to their very high catalytic activity, enzymatic selectivity, ease of synthesis, stability, and low cost, PBNPs are a promising alternative to expensive and insufficiently stable natural peroxidase. Hence, PBNPs are used as labels for the LFIA of different analytes [49,50].

## 2. Materials and Methods

### 2.1. Reagents

The MG and the monoclonal antibodies (Mab) against MG (clone 7C3, Mab7C3) were from Bialexa (Moscow, Russia). The Mab against MG (clone MyoA6, MabA6) were from the Russian Research Center for Molecular Diagnostics and Therapy (Moscow, Russia). The goat anti-mouse polyclonal immunoglobulins (GAMI) were from Arista Biologicals (Allentown, PA, USA). The gold(III) chloride hydrate (HAuCl_4_ × H_2_O), iron(III) chloride hexahydrate (FeCl_3_ × 6H_2_O), potassium(II) hexacyanoferrate trihydrate (K_4_[Fe(CN)_6_] × 3H_2_O), bovine serum albumin (BSA), N-hydroxysulfosuccinimide (sulfo-NHS), 1-ethyl-3-(3-dimethylaminopropyl)carbodiimide (EDC), Triton X-100, Tween 20, citric acid (C_6_H_8_O_7_), sucrose, sodium azide, and sodium citrate were from Sigma-Aldrich (Saint Louis, MO, USA). Peroxidase substrate mixtures with 3,3′,5,5′-tetramethylbenzidine (TMB) or 3,3′-diaminobenzidine (DAB) as the main component were from Immunotech (Moscow, Russia) and ServiceBio (Wuhan, China), respectively. All other compounds of analytical grade were from Khimmed (Moscow, Russia).

### 2.2. Synthesis of AuNPs and PBNPs

AuNPs ~30 nm in diameter were prepared by the reduction of chloroauric acid [51]. Briefly, to 146.25 mL of deionized water, 1.5 mL of a 1% solution of HAuCl_4_ was added, and the resulting mixture was heated to 100 °C. After that, 2.25 mL of a 1% solution of sodium citrate was added with vigorous agitation. The mixture was kept boiling for 25 min. The synthesized AuNPs with optical density (OD) at 520 nm = 1 were stored at 4 °C.

PBNPs were synthesized as described in [52]. Solutions of K_4_[Fe(CN)_6_] and FeCl_3_ (1 mM both) were prepared, and then 98 mg of C_6_H_8_O_7_ was added to each solution (20 mL). The obtained solutions were heated to 60 °C and quickly mixed. After incubation for 3 min at 60 °C, the mixture was stirred until it reached room temperature (RT) (~90 min). The obtained PBNPs were centrifuged for 50 min at 14,500 g, and the sediment was resuspended in 50 mM sodium phosphate buffer at pH 7.4 up to the initial volume.

To characterize AuNPs and PBNPs by size and homogeneity, transmission electron microscopy (TEM) was implemented on a JEM-100C microscope (JEOL, Tokyo, Japan).

### 2.3. Conjugation of AuNPs and PBNPs with Mab

Before conjugation with the AuNPs, a dialysis of МAbA6 against 10 mM Tris-HCl buffer at pH 9.0 was carried out. The pH of the AuNPs was brought to pH 9.0 by adding 0.1 M potassium carbonate. MabA6 (10 μg/mL) was added to the AuNPs (OD_520_ = 1) and stirred for 1 h at RT. Then, 10% BSAwas poured into the reaction mixture to obtain a 0.25% solution and stirred for 15 min. After that, the Mab–PBNPs were centrifuged for 15 min at 12,000 g and 4 °C. The precipitate was dissolved in 0.01 M Tris-HCl with pH 9.0, containing 0.01% sodium azide, 1.0% sucrose, and 1.0% BSA (TBSA) to OD_520_ = 15. The Mab–AuNPs were kept at 4 °C.

To prepare the Mab–PBNPs conjugates, 8 mL of PBNPs solution, 64 mg of sulfo-NHS, and 32 mg of EDC were blended and incubated for 25 min at RT. The MabA6 were added to obtain concentrations of 5, 10, 15, 20, and 50 µg/mL and stirred for 2 h at RT. After that, 10% BSA in 50 mM phosphate buffer with pH 7.4 and containing 0.1 M NaCl (PBS) was added to each Mab/PBNPs mixture to reach a concentration of 0.25%, and was stirred at RT for 15 min. The mixtures were centrifuged at 14,500 g for 15 min, and the sediments were resuspended in PBS (this procedure was repeated three times). Finally, the obtained Mab–PBNPs conjugates (Mab–PBNPs_5_, Mab–PBNPs_10_, Mab–PBNPs_15_, Mab–PBNPs_20_, and Mab–PBNPs_50_) were resuspended in 800 µL of water solution of 1% BSA and 1% sucrose and kept at 4 °C.

### 2.4. Obtaining of Immunochromatographic Test Strips

Two types of test strips were manufactured for AuNPs-based and PBNPs-based assays, respectively. In both cases, a CNPC-SS12 working nitrocellulose membrane of 15 µm pore size and an AP045 adsorption pad (Advanced Microdevices, Ambala Cantt, India) were utilized. For the AuNPs-based LFIA, a GFB-R4 sample pad and a PT-R7 fiberglass conjugate pad (both from the same source) were used. The Mab7C3 (2.5 mg/mL) and GAMI (0.15 and 0.25 mg/mL for the AuNPs- and PBNPs-based LFIAs, respectively) were immobilized at a loading of 0.1 µL/mm to make a test zone (T zone) and a control zone (C zone) on the nitrocellulose membrane using an Iso-Flow dispenser (Imagene Technology, Hanover, NH, USA). The immobilization was carried out in PBS for AuNPs-based LFIA and in PBS with 0.1% sodium azide, 0.25% sucrose, and 0.25% BSA for PBNPs-based LFIA. In the AuNPs-based assay, a Mab–AuNPs conjugate in TBSA containing 0.05% Tween-20 (OD_520_ = 6) was immobilized on the conjugate pad.

All membranes with reactants were kept overnight at RT and for 1.5 h at 37 °C. For the AuNPs-based LFIA, an adsorption pad, a sample pad, and a conjugate pad were fixed on a plastic support. For the PBNPs-based LFIA, no conjugate and sample pads were used; the test strips with a glued adsorption pad were shortened to the bottom edge of the nitrocellulose membrane. The prepared composites were cut by a cutter (KinBio, Shanghai, China) into test strips of 3.2 mm width. After storage with silica gel in sealed packages for at least 2 months at RT, the parameters of the LFIAs did not change.

### 2.5. Sample Preparation

Pork, chicken, lamb meat, and sausages (boiled sausage from pork/beef/pork fat, semi-smoked sausage from pork/beef, and vegan sausage from vegetable raw materials) were purchased in local grocery stores. For sample preparation, the procedure described in our previous study was applied with slight modifications [53]. First, all meat products were minced by a household blender. A homogenized meat sample (250 mg) was added to an extraction buffer (PBS with 1% Tween-20 (PBSTw_1_) containing 0.5 M KCl, 5 mL). Then, the mixtures were shaken for 15 min and sonicated in an ultrasound bath for 15 min for better extraction of MG. The obtained extracts were centrifuged to separate the solid part at 5000 g for 10 min and RT. The upper parts were separated and analyzed by the LFIA.

### 2.6. LFIAs of MG

To perform AuNPs-based LFIA, test strips were placed vertically in MG solutions prepared in PBS containing 0.05% Triton X-100 (PBST) (3333–1.5 ng/mL, 100 μL). After 15-min incubation at RT, test strips were taken out, blotted, and scanned with a CanoScan 9000F scanner (Canon, Tochigi, Tokyo, Japan). The scans were processed by TotalLab TL120 software (Nonlinear Dynamics, Newcastle, UK) to register the intensity of the band coloration (in relative units, RU).

For the PBNPs-based LFIA, test strips were placed vertically in the standard solutions of MG in PBSTw_1_ (3333–0.5 ng/mL, 40 μL) or extracts from meat products (40 μL). After 10 min, the test strips were transferred to a solution of 5% BSA in PBSTw_1_ (40 μL) and kept for another 5 min. Next, the test strips were taken out and placed semi-horizontally, and aliquots of the Mab–PBNPs conjugate (1.5 μL) were dropped onto each strip close to its lower edge. After 5-min incubation, the test strips were placed vertically in PBSTw_1_ (40 μL) and kept for 7 min. For the enhanced LFIA, 1 μL of DAB was applied to the T zone and incubated for 2.5–3 min. The final processing was performed as described above.

To fit the color intensities of the T zones (y) versus MG concentrations (x), OriginPro 9.0 software (OriginLab, Northampton, MA, USA) was utilized. Visual LOD was estimated as the minimum concentration of MG, which caused a visible coloration of the T zone.

## 3. Results and Discussion

### 3.1. Preparation of Key LFIA Reagents and Their Characterization

**Figure 1:**
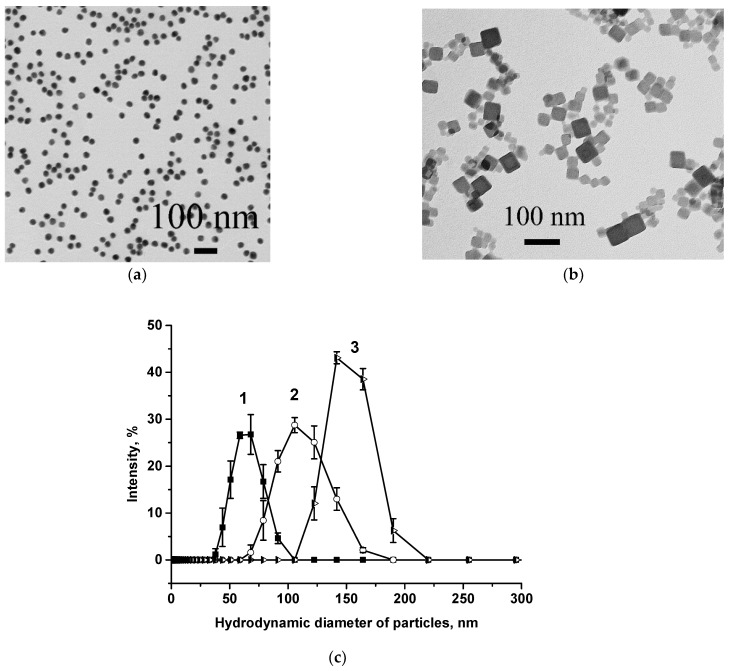
Characterization of AuNPs, PBNPs, and MabA6–PBNPs conjugates by TEM (**a**,**b**) and DLS (**c**); electronic photographs of AuNPs (**a**) and PBNPs (**b**) and the results of DLS (**c**) for PBNPs (1), MabA6–PBNPs_10_ (2), and MabA6–PBNPs_15_ (3) (n = 3).

At the first stage of the work, the key reagents of the test system—the markers and their conjugates with antibodies—were prepared and characterized. Two types of nanoparticles were synthesized as labels: AuNPs (as a traditional label in the LFIA) and PBNPs. AuNPs were prepared by the gold salt reduction, and PBNPs by the reaction of potassium hexacyanoferrate with iron chloride followed by the formation of potassium–iron hexacyanoferrate. The surfaces of the PBNPs contained carboxyl groups due to the citric acid added during synthesis. The resulting markers were observed by TEM for size, shape, and aggregation; the PBNPs were additionally studied by dynamic light scattering (DLS) using Zetasizer Nano ZS 90 (Malvern, UK). According to TEM, the AuNPs were non-aggregated O-shaped nanoparticles with an average diameter of 28.0 ± 3.2 nm (maximum and minimum diameters were 40.3 and 15.9 nm, respectively) and ellipticity of 1.2 ± 0.2 (Figure 1a). The PBNPs preparation contained cubic nanoobjects (Figure 1b) with an average size estimated by DLS of about 60 nm (curve 1 in Figure 1c).

The MabA6 and Mab7C3 used as receptors for MG were conjugated to both labels. For the AuNPs, conjugation was carried out using the adsorption interaction method. The concentration of the MabA6 for complexation with AuNPs (10 μg/mL) was determined in our previous study [31] according to the method of flocculation curves described in [54]. The synthesis of the Mab–PBNPs was carried out in two stages: carbodiimide activation of carboxyl residues on the PBNPs’ surface, and conjugation of the protein with activated particles. For conjugation, five concentrations of Mab in PBS were used: 5, 10, 15, 20, and 50 μg/mL. The attachment of antibody molecules to PBNPs may lead to aggregation of the latter, proportional to protein concentration [52]. The addition of Mab at high concentrations (20 and 50 μg/mL) to the PBNPs caused their precipitation. Therefore, Mab–PBNPs_20_ and Mab–PBNPs_50_ conjugates were excluded from the consideration. DLS data on the Mab–PBNPs conjugates demonstrated a peak shift towards increasing particle average size from ~60 nm (unmodified PBNPs, curve 1 in Figure 1c) to 105 and 165 nm for Mab–PBNPs_10_ and Mab–PBNPs_15_, respectively (curves 2, 3 in Figure 1c), which may be evidence of possible aggregation while maintaining colloidal stability.

### 3.2. Principle of the LFIA

The immunochromatographic detection of porcine MG was implemented in a sandwich scheme dominated for the detection of polyvalent high molecular weight analytes as a more sensitive and selective assay due to the recognition of multiple epitopes. To implement the LFIA, specific Mab7C3 antibodies were immobilized in the T zone, and anti-species GAMI were absorbed in the C zone. If porcine MG is present in the sample, it interacts with a Mab–label conjugate and the resulting complex binds to Mab immobilized in the T zone of the test strip. A ternary Mab–MG–Mab–label complex is formed and the first colored band is observed. In this case, the coloration intensity reflected the analyte concentration. The excess of labeled antibodies moved further and bound with anti-species antibodies in the C zone, which resulted in the formation of the second colored line. If there was no porcine MG in the sample, only the C zone became colored (the upper band is visualized).

To evaluate the gain in the assay sensitivity when using catalytic amplification, MG determination was carried out using both the AuNPs label and a nanozyme one with colorimetric detection. The enhanced LFIA of MG is based on the catalytic properties of a label that mimics the enzymatic activity of peroxidase. PBNPs as a pigment have a bright blue color and can be used as a colorimetric marker. The addition of peroxidase substrate to blue-colored test strips after the LFIA initiated the catalysis of the reaction oxidizing the substrate to an insoluble product, whose brown color contributed to the overall signal intensity. Therefore, catalytic amplification of the signal occurred, increasing the assay sensitivity (it became possible to reliably visualize bands that were poorly visible or not at all noticeable after the LFIA without amplification).

### 3.3. AuNPs-Based LFIA of MG

**Figure 2:**
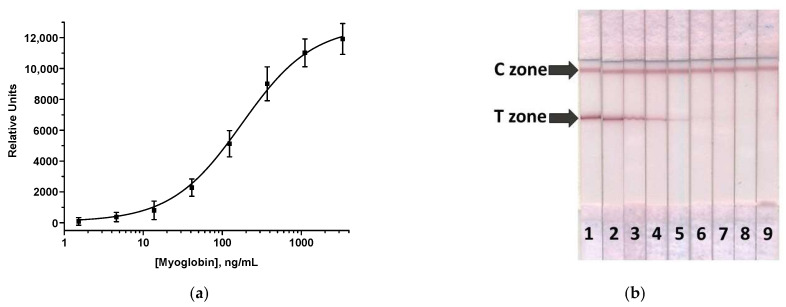
Calibration curve of MG in the AuNP-based LFIA (**a**) and images of test strips (**b**). The numbers on the test strips reflect the concentrations of MG in the sample (ng/mL): 3333 (1), 1111 (2), 370 (3), 124.5 (4), 41.2 (5), 13.7 (6), 4.6 (7), 1.5 (8), and 0 (9) (n = 3). The zones are indicated by arrows.

The LFIA with AuNPs was carried out in the simplest one-step format, where all reagents were pre-applied to the test strip, and the assay consisted of incubating the sample with the strip. The analytical procedure was optimized to reach the minimum MG LOD and the coloration intensity sufficient for accurate and reproducible determination of MG. To do this, the concentrations of immobilized antibodies and labeled Mab in solution and the assay duration were varied (Table S1). Thus, the Mab7C3 and GAMI concentrations were changed in the ranges of 0.5–3 mg/mL and 0.1–0.5 mg/mL, respectively; the OD_520_ of MabA6–AuNPs conjugate was varied from 2 to 8. It was shown that the best analytical characteristics were achieved when Mab7C3 and GAMI were immobilized at concentrations of 2.5 and 0.15 mg/mL, the concentration of MabA6–AuNPs corresponded to OD_520_ = 6, and the incubation time was 15 min. In the optimized conditions, the visual LOD of porcine MG was 13.7 ng/mL; the corresponding calibration curve and the appearance of test strips after the LFIA are presented in Figure 2.

### 3.4. Enhanced LFIA of MG

#### 3.4.1. Extinguishing of Non-Specific Interactions

In the first step, test strip composition was selected. The standard test strips created from a multicomposite with sample, adsorption, and conjugate pads fixed on the adhesive support with a working membrane were used. Test strips were applied to samples with no MG (zero point) and at MG concentration of 10 μg/mL. As a result, it was shown that all labeled antibodies adhered at the bottom of the strip and scarcely moved with the liquid flow; thus, no coloration of the T and C zones was observed (Figure S1a). To eliminate this obstacle, the test strips were cut to the lower edge of the working membrane, and thus sample and conjugate pads were excluded from their composition. Accordingly, the one-stage sandwich format was transformed into a two-stage one, where the analyzed sample was pre-incubated with Mab–PBNPs and subsequently incubated with the test strip. With this configuration of the test system, the labeled Mab moved much more effectively along the working membrane and interacted in the zones, which accordingly turned blue due to the PBNPs. However, in these conditions, non-specific binding in the T zone was observed at the zero point (Figure S1b). Non-specific adsorption of PBNPs is described in other works, for example in [52], where the authors elaborated methods to extinguish such binding. In our study, various approaches were also tested, including the choice of a medium for antibody sorption, pretreatment of the working membrane, varying the sequence of interactions, etc. First, a medium with a high detergent content was tested for the interaction. This approach was undertaken both to eliminate non-specificity and to ensure good mobility of the reagents along the membrane. PBS-based media were tested with detergents Tween-20 and Triton X-100 at content levels 2 and 20 times higher than those in commonly used PBST (namely, 0.1 and 1%.) The movement of reagents and leaching of labeled antibodies from the membrane carrier were most effectively achieved when using PBSTw_1_; therefore, this medium was used for further experiments. Secondly, the immobilization of the reagents onto the working membrane was carried out in a special medium containing 0.1% sodium azide, 0.25% BSA, and 0.25% sucrose.

**Figure 3:**
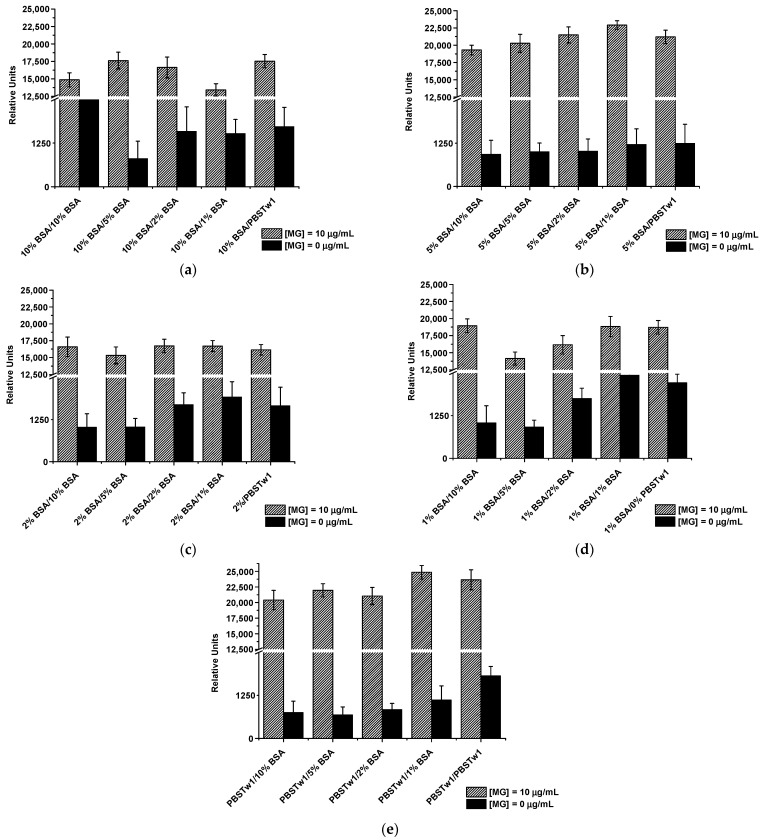
Results of the selection of the media for interactions during Step 1 (dashed columns) and blocking agent during Step 2 (filled columns) for the working membrane. Media for interactions were 10% BSA (**a**), 5% BSA (**b**), 2% BSA (**c**), 1% BSA (**d**), and PBSTw_1_ (**e**).; blocking agents were 10% BSA, 5% BSA, 2% BSA, 1%BSA, and PBSTw_1_ in every scheme.

Because non-specific interactions were observed in the two-stage sandwich assay format, another scheme with sequential interaction of immunoreagents (four-stage scheme) was proposed. In this mode, the following sequence of actions was implemented: incubation of the test strips with the sample (1) → incubation of the test strips with a blocking buffer (2) → incubation with labeled Mab (3) → washing of the strip with the buffer (4). Several variants of interaction media and blocking agents for processing test strips were tested, where PBSTw_1_ or solutions with different BSA contents (1, 2, 5, and 10% in PBSTw_1_) were used. The results of the LFIA for various combinations are presented in Figure 3. The criterion for the applicability of a particular combination was the minimum signal intensity at the zero point (at ~600–700 RU the zones could not be visualized by the naked eye) and the maximum signal difference at MG concentrations of 0 and 10 μg/mL. According to the data obtained, the medium for interactions at stage (1) did not play a significant role in suppressing non-specificity in contrast to the medium used to block the membrane at stage (2). Thus, when performing stage (1) in PBSTw_1_ with 10, 5, 2, and 1% BSA, signal changes at 10 μg/mL MG concentration were negligible (Figure 3a–d). Moreover, the addition of BSA to a sample is implementable when analyzing MG in a model system (buffer) but is not desirable during the analysis of real samples. Therefore, step (1) was further carried out in PBSTw_1_. As can be seen from Figure 3e, the absence of blocking by BSA led to an almost threefold increase in the signal at the zero point. Therefore, the membrane blocking mode with a minimum BSA content (namely, 5%) was chosen, ensuring the suppression of non-specificity.

#### 3.4.2. Selection of the Labeled Conjugate

**Figure 4:**
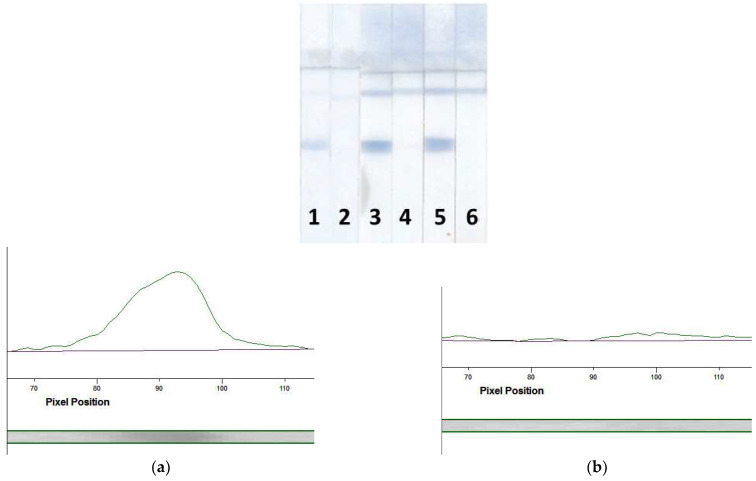

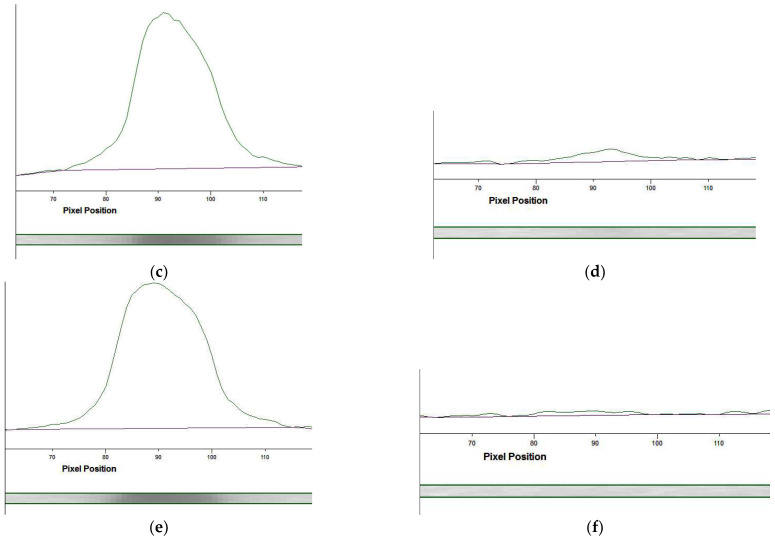
Test strips after the LFIA of MG using Mab–PBNPs_5_ (strips 1 and 2), Mab–PBNPs_10_ (strips 3 and 4), and Mab–PBNPs_15_ (strips 5 and 6) conjugates. Coloration intensity profiles of T zones at MG concentrations of 10 ng/mL (profiles (**a**,**c**,**e**) and 0 ng/mL (strips (**b**,**d**,**f**)).

For the LFIA, three labeled conjugates were tested: Mab–PBNPs_5_, Mab–PBNPs_10_, and Mab–PBNPs_15_. All conjugates interacted with the detected MG providing the coloration of the T and C zones (Figure 4). The coloration intensity of the Mab–PBNPs_5_ conjugate was lower than those of the Mab–PBNPs_10_ and Mab–PBNPs_15_ (compare test strips 1, 3, and 5 and profiles a, c, and e in Figure 4), but the Mab–PBNPs_15_ conjugate was not stable enough: the nanoparticles precipitated approximately one week after the synthesis and therefore could not be used for the assay development, although it provided a high analytical signal before aggregation. Therefore, the Mab–PBNPs_10_ were chosen for subsequent enhanced LFIA of porcine MG.

#### 3.4.3. Optimization of the LFIA

Following all the obtained regularities, the LFIA for the detection of MG with PBNPs was implemented. First, the detection was carried out using the colorimetric properties of the pigment. Assay optimization included the choice of free and immobilized reactant concentrations and the times of sequential stages in the sandwich analysis (Table S1). It was shown that the best assay performance was ensured if the concentration of the immobilized Mab7C3 was 2.5 mg/mL (a lesser amount resulted in a decrease in the bands’ brightness, and a greater amount did not affect the intensity, but caused a greater expenditure of reagents). The GAMI concentration in the C zone was 0.25 mg/mL (the reasons are the same as those described above). The optimal volume of the labeled antibodies was 1.5 μL; increasing this amount led to a slight increase in the signal intensity, but also the emergence of the background coloration of the working membrane. A lower volume of labeled antibodies led to a decrease in the signal intensity.

**Figure 5:**
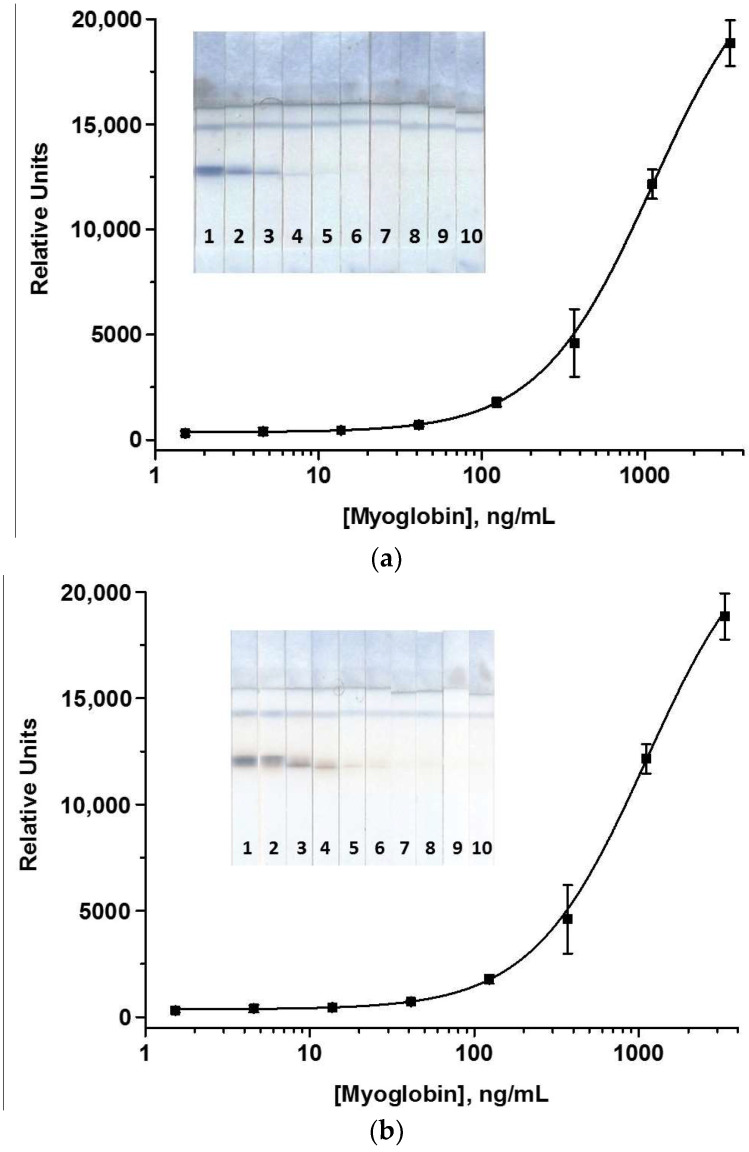
Calibration curves of MG and images of the test strips in the PBNPs-based LFIA with no signal amplification (**a**) and in the enhanced LFIA (**b**). The numbers on the test strips indicate the concentrations of MG in the sample (ng/mL): 3333 (1), 1111 (2), 370 (3), 124.5 (4), 41.2 (5), 13.7 (6), 4.6 (7), 1.5 (8), 0.5 (9), and 0 (10) (n = 3).

Lastly, the duration of stages (1)–(4) of the LFIA was optimized. Stage (1) was carried out for 10 min; this time was quite sufficient to absorb 40 μL of the detected sample with the shortened test strip and for the progress of all immunochemical reactions. Longer incubation only increased the total assay time. The second stage was carried out within 5 min, because no interactions occurred during it, and only the blocking of non-specific binding centers was required. The third stage was also carried out for 5 min, within which a very small volume of labeled Mab (1.5 μL) was distributed near the T zone only. To enable their further movement, Stage (4) was necessary, which involved washing the test strip with buffer. To effectively wash off the labeled Mab from the test strip and promote their reaction in the zones, this stage was carried out for 7 min. The MG calibration curve in the optimized PBNPs-based LFIA without amplification and the scans of corresponding test strips are presented in Figure 5a. The assay was characterized by a visual LOD of 40 ng/mL and was able to be implemented within 27 min.

The enhanced LFIA of MG involving the catalytic properties of PBNPs was carried out under the conditions (concentrations of reagents and duration of stages) selected above. Two compounds, TMB or DAB, were used as chromogenic substrates for oxidation catalyzed by PBNPs. They were applied to the test strips after the analytical procedure. It should be noted that TMB caused the appearance of a non-specific signal at the zero point (~2500 RU) and background coloration of the whole working membrane (Figure S2). In the case of DAB, these shortcomings were absent, so it was used in further analysis. However, the volume of the applied substrate should be the minimum required for coloration only of the T zone, to avoid uneven spreading over the working membrane and the occurrence of local stains, which significantly complicates the adequate processing of scanned test strips. This was achieved by applying 1 μL of DAB. The insoluble product of the DAB reaction had a brown tint and made an additional contribution to the zones’ brightness. Coloration developed gradually and reached a maximum at approximately 2.5–3 min of incubation with the substrate. A further prolongation of the catalytic reaction led to the development of a background coloration. The calibration curve for the enhanced LFIA using nanozyme and the image of test strips are presented in Figure 5b. According to the data obtained, the visual LOD of porcine MG was 1.5 ng/mL. Therefore, the 9-fold and 27-fold gains in the sensitivity were achieved as compared with the AuNPs-based LFIA and LFIA with PBNPs in the non-enhanced mode.

### 3.5. Determination of MG in Meat Samples

**Figure 6:**
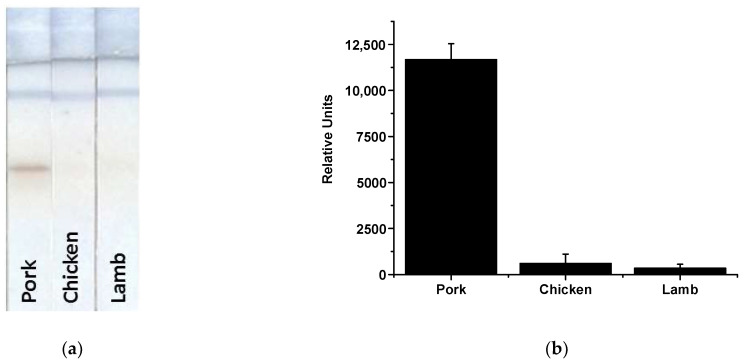
Images of test strips after the enhanced LFIA in meat extracts (**a**) and the corresponding analytical signals (**b**).

The successful development of the enhanced LFIA of MG enabled its use for biomarker detection in real samples. The latter included samples of raw meat (pork, chicken, and lamb) and finished meat products—boiled pork/beef/pork fat sausage (sample 1), semi-smoked pork/beef sausage (sample 2), and vegan sausage (sample 3). Before the analysis, meat samples were processed to remove the matrix and effectively extract MG. To achieve this, a technique consisting of extraction with a special buffer followed by separation of the liquid part to be analyzed by centrifugation was used. We took into account all the patterns identified during test system elaboration, including conditions for the suppression of non-specific interactions and used an extractant based on PBSTw_1_. The results of MG detection in the extracts from pork, lamb, and chicken are shown in Figure 6. According to these results, the test system detected only porcine MG (T zone coloration is visualized only as a result of analyzing the pork extract).

**Figure 7:**
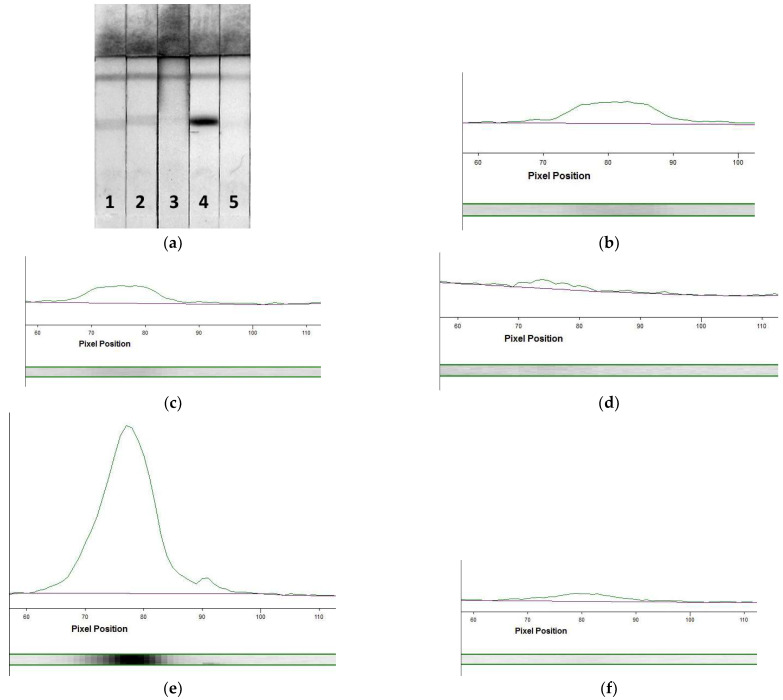
Processing of test strips. (**a**) Scans of test strips after the enhanced LFIA of sample 1 (test strip 1), sample 2 (test strip 2), sample 3 (test strip 3), MG with the concentration of 2 mg/mL (test strip 4), and 0 mg/mL (test strip 5); (**b**–**f**)—coloration intensity profiles of T zones of the test strips 1 (**b**), 2 (**c**), 3 (**d**), 4 (**e**), and 5 (**f**).

Data obtained from the determination of MG in extracts of finished meat foodstuffs are presented in Figure 7. As can be seen from the scans of the test strips (Figure 7a), T zone coloration occurs only as a result of the analysis of extracts from sausages containing pork (strips 1 and 2). Analysis of the extract from vegan sausage (test strip 3) did not reveal MG (the results of the LFIA of solutions containing MG at concentrations of 2 µg/mL (test strip 4) and 0 mg/mL (test strip 5) are presented as positive and negative controls). Figure 7b–f gives the profiles of the coloration intensities in the enhanced LFIA of the above-indicated extracts/MG solutions. A significant difference between the signal at the zero point (profile f) and sample 1 (profile b), sample 2 (profile c), and MG at the concentration of 2 µg/mL (profile e) was confirmed.

### 3.6. Comparison with Other Studies

Because PBNPs-based nanozymes were not previously used to develop the LFIA of species-specific markers, including MG, it is not possible to fully compare the obtained results with the earlier ones. PBNPs as a label for the LFIA were used in several works [52,55,56,57,58,59,60,61,62,63] aimed at the detection of different analytes—from low molecular weight haptens (mycotoxins, drugs, disease biomarkers) to biomolecules (antibodies) and bacterial cells (Table 1).

In some works, the peroxidase-like catalytic action of PBNPs was not applied and the common colorimetric detection of blue pigment was carried out, which allowed LODs of zearalenone [56] and clenbuterol [57] to be reduced by five times compared to AuNPs-based LFIAs. In [55], an original approach based on the fading of PBNPs by NaOH with no catalytic enhancement was used. The applied technique enabled the LOD of aflatoxin B1 to be reduced by eight times. In the majority of studies dealing with the PBNPs-based enhanced LFIA, no comparison with the traditional AuNPs-based assay has been made. Only in two papers did the authors estimate the gain in sensitivity of the enhanced analysis; it was tenfold for β-agonists detection [59] and two to three orders of magnitude for rabbit IgG detection [52]. The described LFIAs were carried out in a buffer [61] and in such real samples as urine, serum, cereals, and meat extracts (other studies). The time of analysis varied in the range of 2–30 min. In our study, the use of nanozyme enhancement enabled a 9-fold and 27-fold reduction in the LOD of MG in comparison with AuNPs-based and PBNPs-based colorimetric assays, exceeding the average gains reported in other studies. The assay was able to be finished within 30 min, including the catalytic stage, and thus this method is rapid. It should be noted that although the LOD of MG was significantly reduced by the proposed approach, the multi-step mode of the LFIA was accompanied by a complication of the technique as a whole. In this regard, a prospective direction of our development is a transition to a test system with pre-applied substrate components, although the selection of such peroxides characterized by long-term storage without decomposition requires additional study.

## 4. Conclusions

An immunosensor aimed at the analysis of pork MG was designed based on the enhanced LFIA with a nanozyme label. This highly sensitive, rapid, and specific analysis may serve as a promising method for accurate and rapid pork authentication in raw materials and semi-finished and finished meat foodstuffs (including thermally processed ones). The described approach will contribute to ensuring the quality of food products and help promote policies to guarantee consumers can purchase high-quality products and to provide them with a healthy diet. The LFIA of MG can be used at any stage of the production and sale of meat products—from food industry enterprises to home and public catering outlets.

## Figures and Tables

**Table 1:** Studies on the LFIAs using PBNPs.

Analyte	Compound for Amplification (If Used)	Assay Duration, min	LOD	Gain in Sensitivity Compared to AuNP-Based LFIA	Reference
Rabbit IgG/ochratoxin A	TMB	30	0.01 ng/mL/10 ng/mL	2–3 orders/n.p. *	[52]
Aflatoxin B1	NaOH	28	0.023 ng/mL	8-fold	[55]
Zearalenone	n.u. **	6	10 µg/kg	5-fold	[56]
Clenbuterol	n.u.	15	1 ng/mL	5-fold	[57]
Glycocholic acid	TMB	10	10 ng/mL	n.p.	[58]
β-Agonists	DAB	20	0.3–0.5 μg/kg	10-fold	[59]
*Brucella* antibodies	TMB	2–3	40 IU/mL	n.p.	[60]
Ractopamine/ clenbuterol	TMB	10	6/12 ng/mL	n.p.	[61]
*Escherichia coli* O157:H7	TMB	25	10^2^ CFU/mL	n.p.	[62]
Uric acid	TMB	n.p.	Detection range = 15–85 μg/mL	n.p.	[63]
MG	DAB	30	1.5 ng/mL	9-fold	This study

* n.p.—not presented. ** n.u.—not used.

## Data Availability

The data used to support the findings of this study can be made available by the corresponding author upon request.

## Supplementary Materials

The following supporting information can be downloaded at: https://www.mdpi.com/article/10.3390/foods12234252/s1. Figure S1: Images of test strips after the LFIA of MG at concentrations of 10 µg/mL (strips 1, 3, and 5) and 0 µg/mL (strips 2, 4, and 6). For the assay, conjugates of Mab–PBNPs_15_ (strips 1 and 2), Mab–PBNPs_10_ (strips 3 and 4), and Mab–PBNPs_5_ (strips 5 and 6) were used. Stacking of labeled Mab at the bottom of the test strip (**a**); non-specific binding at zero point (**b**). Figure S2: Calibration curve of MG after the enhanced LFIA with TMB as a substrate and images of the test strips (**a**). The numbers on the test strips reflect the concentrations of MG in the sample (ng/mL): 3333 (1), 1111 (2), 370 (3), 124.5 (4), 41.2 (5), 13.7 (6), 4.6 (7), 1.5 (8), 0.5 (9), and 0 (10) (n = 3). Signal intensities at concentrations of MG of 0 and 1000 ng/mL after the enhancement using TMB and DAB (**b**). Table S1: Parameters optimized during LFIA development.
