# Supplementary material for: Highly Sensitive Immunochromatographic Detection of Porcine Myoglobin as Biomarker for Meat Authentication Using Prussian Blue Nanozyme
Source: Foods. 2023 Nov 24;12(23):4252. doi: 10.3390/foods12234252 (PMC10706380; doi:10.3390/foods12234252)
Supplement: Supplementary file 1 [file foods-12-04252-s001.zip › foods-2708829-supplementary.pdf]

foods

Article

Highly Sensitive Immunochromatographic Detection of Porcine Myoglobin as Biomarker for Meat Authentication Using Prussian Blue Nanozyme

Olga D. Hendrickson, Elena A. Zvereva, Boris B. Dzantiev and Anatoly V. Zherdev *

A.N. Bach Institute of Biochemistry, Research Center of Biotechnology of the Russian Academy of Sciences, Leninsky prospect 33, 119071 Moscow, Russia; odhendrick@gmail.com (O.D.H.); zverevaea@yandex.ru (E.A.Z.); dzantiev@inbi.ras.ru (B.B.D.)

* Correspondence: zherdev@inbi.ras.ru; Tel.: +7-495-954-28-04

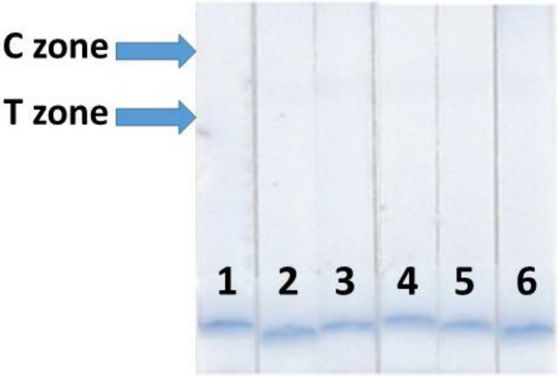

(**a**)

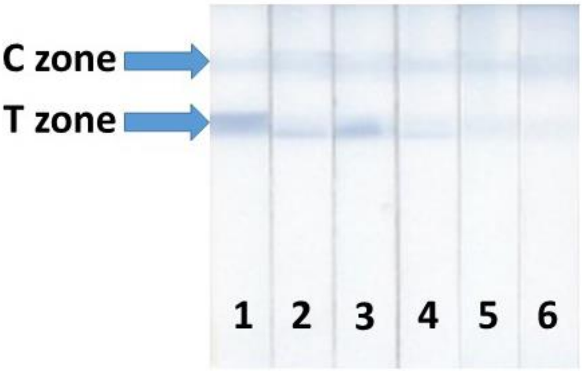

(**b**)

Figure S1. Images of test strips after the LFIA of MG at concentrations of 10 μg/mL (strips 1, 3, and 5) and 0 μg/mL (strips 2, 4, and 6). For the assay, conjugates of Mab–PBNPs$_{15}$ (strips 1 and 2), Mab–PBNPs$_{10}$ (strips 3 and 4), and Mab–PBNPs$_{5}$ (strips 5 and 6) were used. Stacking of labeled Mab at the bottom of the test strip (**a**); non-specific binding at zero point (**b**).

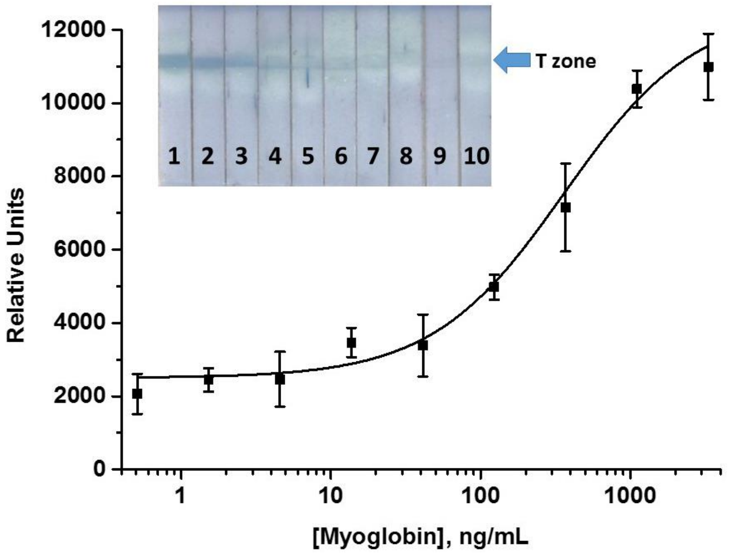

(**a**)

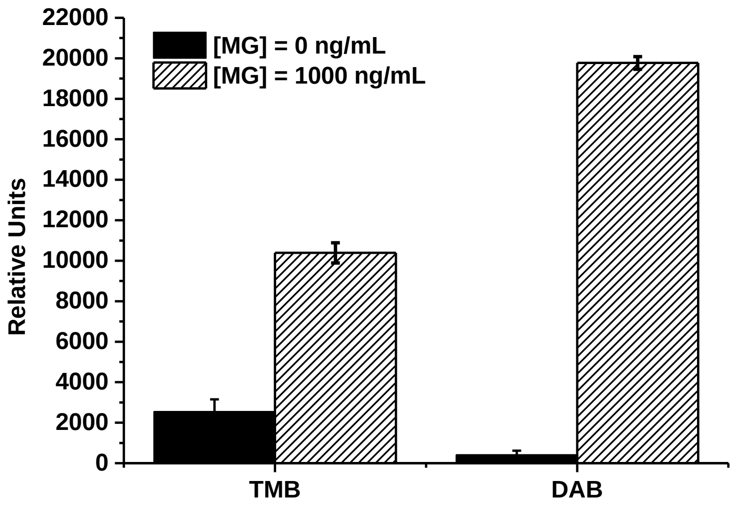

(**b**)

Figure S2. LFIA with TMB as a substrate and images of the test strips. (**a**) The numbers on the test strips reflect the concentrations of MG in the sample (ng/mL): 3333 (1), 1111 (2), 370 (3), 124.5 (4), 41.2 (5), 13.7 (6), 4.6 (7), 1.5 (8), 0.5 (9), and 0 (10) (n = 3). Signal intensities at concentrations of MG of 0 and 1000 ng/mL after the enhancement using TMB and DAB (**b**).

Table 1. Parameters optimized during LFIA development.

Parameter	Range of Variation	Selected Variant
**AuNPs-based LFIA**		
Concentration of the immobilized Mab7C3, mg/mL	0.5–3	2.5
Concentration of the immobilized GAMI, mg/mL	0.1–0.5	0.15
OD_{520} of the MabA6–AuNPs conjugate	2–8	6
Assay duration, min	10–20	15
**PBNPs-based LFIA**		
Concentration of the immobilized Mab7C3, mg/mL	0.5–3	2.5
Concentration of the immobilized GAMI, mg/mL	0.1–0.5	0.25
Volume of the MabA6–AuNPs conjugate, μL	1–7.5	1.5
Duration of stage 1, min	5–15	10
Duration of stage 2, min	3–10	5
Duration of stage 3, min	3–10	5
Duration of stage 4, min	5–10	7
Duration of catalytic reaction, min	1.5–5	3
